# Bu Shen Zhu Yun Decoction Improves Endometrial Receptivity via VEGFR-2-Mediated Angiogenesis

**DOI:** 10.1155/2019/3949824

**Published:** 2019-12-31

**Authors:** Li Li, Huabo Jiang, Xuecong Wei, Dandan Geng, Ming He, Huilan Du

**Affiliations:** ^1^Hebei Key Laboratory of Integrative Medicine on Liver-Kidney Patterns, College of Integrative Medicine, Hebei University of Chinese Medicine, Shijiazhuang, China; ^2^Department of Gynecology, Shanghai First Maternity and Infant Hospital Affiliated to Tongji University School of Medicine, Shanghai, China

## Abstract

Vascular endothelial growth factor receptor-2 (VEGFR-2) regulates the mitogen-activated protein kinase (MAPK) signaling pathway and plays an important role in angiogenesis. Bu Shen Zhu Yun decoction (BSZYD) can improve endometrial receptivity and embryo implantation rates in patients undergoing in vitro fertilization. However, whether BSZYD improves endometrial receptivity via angiogenesis remains unclear. Here, we investigated the effects of BSZYD on the proliferation, migration, and angiogenesis of human endometrial microvascular endothelial cells (HEMECs) and found that BSZYD upregulated the expression of cyclin D1, matrix metalloproteinase 9 (MMP9), and proliferating cell nuclear antigen (PCNA) in HEMECs. Cell Counting Kit 8 assay, scratch-wound assay, and Tube Formation Assay results showed that BSZYD promoted the proliferation, migration, and angiogenesis of HEMECs. Western blot analysis results revealed the activation of the MAPK signaling pathway by BSZYD through the upregulation of VEGF and VEGFR-2 expression. Together, these findings highlight the novel mechanism underlying BSZYD-mediated improvement in endometrial receptivity through the MAPK signaling pathway.

## 1. Introduction

Implantation is a critical step in pregnancy, and good endometrial receptivity is an important factor for implantation. Embryo implantation is a continuous dynamic process, during which the womb environment undergoes a series of changes. In particular, the endometrium apical surface undergoes several morphological, molecular, and biochemical changes to provide a favorable environment for embryo implantation [1, 2].

Angiogenesis is one of the important biological events and may be associated with endometrial receptivity in the uterus and ovaries of adult women during the reproductive cycle and pregnancy [3]. Considering the periodic proliferation of endometrial vascular system during embryo implantation and development [4], endometrial angiogenesis is regulated by several vasoactive substances and angiogenic factors. Vascular endothelial growth factor (VEGF) is one of the most important angiogenesis regulating factors that is rapidly activated in the preimplantation blastocyst as well as in response to endometrial contact and leads to angiogenesis to ensure survival of the embryo [5]. In general, vascular endothelial growth factor receptor-2 (VEGFR-2/KDR) is considered as the most important VEGF receptor in the process of angiogenesis and known to regulate endometrial angiogenesis [6]. Mitogen-activated protein kinase (MAPK) signaling pathway, located downstream of VEGFR-2, can regulate the proliferation and migration of vascular endothelial cells by a series of cascade reactions [7].

Although assisted reproductive technology (ART) has made a considerable progress in recent years, implantation failure is a common problem affecting the outcome of ART. At present, no effective strategies are known to solve this problem [8, 9]. In China, traditional Chinese medicine (TCM) is often applied as a complementary medicinal approach in patients undergoing in vitro fertilization (IVF). TCM exhibits unique advantages and balances the physiological environment. However, the mechanism of action of TCM on improvement in endometrial receptivity and increased embryo implantation rate is incompletely understood [10, 11].

In the present study, we used Bu Shen Zhu Yun decoction (BSZYD) to improve the endometrial receptivity for implantation. Here, we show that BSZYD promoted the expression of proliferating cell nuclear antigen (PCNA), cyclin D1, matrix metalloproteinase 9 (MMP9), VEGF, and VEGFR-2 in human endometrial microvascular endothelial cells (HEMECs) through the activation of the MAPK signaling pathway. Our results may provide evidence regarding the beneficial effects of BSZYD on endometrial receptivity in ART.

## 2. Materials and Methods

### 2.1. Culture of HEMECs

HEMECs (ScienCell, USA) were incubated at 37°C and 5% CO_2_ in a 10% fetal bovine serum ECM medium, and the culture medium was replaced every other day until the cells reached approximately 90% confluency.

### 2.2. Preparation of Serum-Containing Drugs

Four female volunteers (20–30 years old) with good health and regular menstruation were selected as the test group. The subjects signed the informed consent form and took BSZYD orally for 4 days as instructed. On day 4, venous blood was collected in the morning 1 h after the last dose, and left at room temperature for 2 h. The supernatant was collected after centrifugation at 3,000 rpm for 10 min, filter sterilized with 0.22 *μ*m sterile filters, and stored at −20°C. In addition, four female volunteers (age 20–30 years old) in good health and menstrual regularity were selected as the control group. Venous blood was collected, as per the above method. The composition of Bu Shen Zhu Yun decoction was as follows: shu di (*Rehmannia glutinosa*), dang gui (*Angelica sinensis*), shan yao (*Dioscorea oppositifolia*), shan yu rou (*Cornus officinalis*), gou qi zi (*Lycium chinense*), yin yang huo (*Epimedium brevicornum*), huang qi (*Astragalus membranaceus*), zi he che (*Placenta hominis*), and xiang fu (*Cyperus rotundus*) (information of the nine herbs is shown in Table 1).

### 2.3. Cell Counting Kit-8 (CCK-8) Proliferation Assay

Cell proliferation was evaluated with the CCK-8 (Dojindo, Kumamoto, Japan) assay according to the manufacturer's recommendations. About 100 *μ*L (1 × 10^4^) of cell suspension was seeded into 96-well plates for 2–4 h, and treated with CCK-8 reagent (10 *μ*L/well) for 5 h. The absorbance at 450 nm wavelength was determined with an automated microplate reader (Versa Max, Santak, USA).

### 2.4. Scratch-Wound Assay

HEMECs were inoculated at 5 × 10^5^ cells/well in six-well plates. After reaching 80% confluency, the cell layer was scratched with the tip of a 200 *μ*L pipette. Drugs were separately added to the serum-free medium. Images were captured with a digital camera (BX51T-PHD-J11, Olympus, JPN).

### 2.5. Endothelial Cell Tube Formation Assay

Ice-cold Matrigel was added to the wells of 24-well plates and the plates were incubated at 37°C for 40 min. About 500 *μ*L of 5 × 10^4^ cell suspension was seeded into these plates. The changes in cell morphology were captured with a digital camera (BX51T-PHD-J11, Olympus, JPN). All experiments were performed in quadruplicates, and data are expressed as the length of network (mean length/field).

### 2.6. Western Blot Analysis

HEMECs were lysed in radioimmunoprecipitation assay (RIPA) lysis buffer containing phenylmethylsulfonyl fluoride (PMSF), and the lysates were harvested by centrifugation at 12,000 rpm for 15 min at 4°C. Protein concentrations were determined with bicinchoninic acid (BCA) protein assay (Thermo Fisher Scientific, USA). Equal amounts of protein samples were electrophoresed on 10% sodium dodecyl sulfate polyacrylamide gel electrophoresis (SDS-PAGE) gels and transferred onto polyvinylidene fluoride (PVDF) membranes (Millipore, MA). The membranes were blocked with 5% dry milk in TTBS for 2 h at room temperature and incubated overnight at 4°C with primary antibodies. Following incubation, the membranes were probed with a secondary antibody at room temperature for 1 h, and protein bands were visualized with Image Quant LAS 4000 (GE Healthcare, USA). The antibodies used included antiglyceraldehyde 3-phosphate dehydrogenase (GAPDH) (Proteintech Group, USA), anti-*β*-actin (Proteintech Group, USA), anti-PCNA (Cell signaling technology, USA), anti-cyclin D1 (Abcam, USA), anti-MMP9 (Proteintech Group, USA), anti-VEGF (Epitomics, USA), anti-VEGFR-2 (Arrigo, Taiwan, CHN), anti-extracellular signal-regulated kinase (ERK; Proteintech Group, USA), anti-p-ERK (Proteintech Group, USA), anti-P38 (Proteintech Group, USA), anti-p-P38 (Cell Signaling Technology, USA), anti-c-Jun N-terminal kinase (JNK) (Cell Signaling Technology, USA), and anti-p-JNK (Cell Signaling Technology, USA).

### 2.7. RNA Preparation and Quantitative Real-Time PCR

Total RNA was extracted with TRIzol (Invitrogen), and 600 ng of RNA was subjected to reverse transcription using first-strand cDNA synthesis kit (Vazyme, USA) according to the manufacturer's instructions. Real-time PCR analysis was performed with ABI 7500 FAST system using the AceQ qPCR SYBR Green Master Mix Kit (Vazyme, USA), as per the manufacturer's instructions. Target mRNA levels were normalized against GAPDH level. Target mRNA expression was analyzed using the 2^−ΔΔCt^ method (sequences shown in Table 2).

### 2.8. Statistical Analysis

All statistical analyses were performed using the SPSS21.0 software, and the data are presented as the mean ± standard deviation (SD). One-way analysis of variance and least significant difference tests were used. *P* < 0.05 was considered statistically significant.

## 3. Results

### 3.1. VEGF Activated the MAPK Signaling Pathway and Promoted Angiogenesis in HEMECs by Binding to VEGFR-2

**Figure 1:**
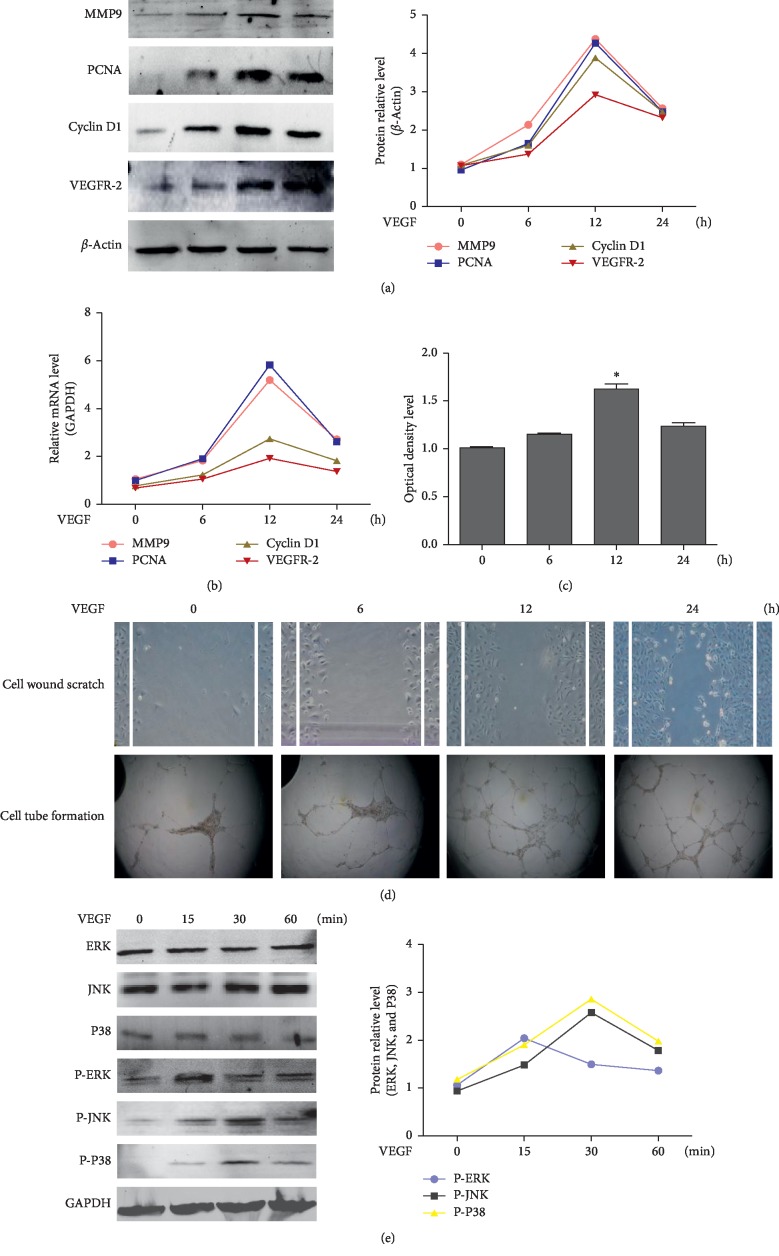
VEGF activated the MAPK signaling pathway and promoted HEMEC angiogenesis by binding to VEGFR-2. (a) HEMECs were incubated in low-serum medium for 24 h and treated with VEGF (40 ng/mL) for indicated time points (0, 6, 12, and 24 h). The expression of MMP9, PCNA, cyclin D1, and VEGFR-2 was determined with western blotting using specific antibodies (left panel). Densitometric scanning (right panel). Values are expressed as the mean ± SD of three independent experiments. (b) MMP9, PCNA, cyclin D1, and VEGFR-2 mRNA level analysis with real-time PCR. Values are expressed as mean ± SD from three independent experiments. (c) The OD level analysis of four groups from CCK-8 assay. Values are expressed as mean ± SD from three independent experiments. ^*∗*^*P* < 0.05 as compared with 0 h group. (d) Images show HEMEC scratch-wound and tube formation assays. Magnification, ×100. (e) HEMECs were incubated in low-serum medium for 24 h and treated with VEGF (40 ng/mL) for indicated times (0, 15, 30, and 60 min). Phospho-ERK, phospho-JNK, phospho-P38, total ERK, total JNK, and total P38 levels were determined with western blotting using specific antibodies (left panel). Densitometric scanning (right panel). Values are expressed as mean ± SD from three independent experiments.

To evaluate the effects of VEGF on HEMECs, we treated these cells with VEGF for different time durations (0, 6, 12, and 24 h) and performed western blot and RT-qPCR analyses. As a result, we found that the expression levels of VEGFR-2, PCNA, cyclin D1, and MMP9 increased in a time-dependent manner and reached peak values at 12 h (*P* < 0.05). The highest levels were maintained until 24 h (Figures 1(a) and 1(b)). CCK-8 assay results showed that VEGF induced cell proliferation (Figure 1(c)). The results of the scratch-wound assay showed that wound healing was significantly faster in VEGF-stimulated cells than in untreated cells (Figure 1(d)), indicating that VEGF promotes HEMEC migration. Endothelial cell tube formation assay results revealed that VEGF induced the angiogenesis of HEMECs (Figure 1(d)). To evaluate the effects of VEGF on MAPK signaling pathway, we treated HEMECs with VEGF for different time durations (0, 15, 30, and 60 min), and performed western blot analysis to detect the changes in MAPK signaling molecules. As a result, we found that VEGF markedly stimulated ERK phosphorylation within 15 min as compared with the untreated groups (*P* < 0.05), and the expression levels of p-JNK and p-P38 increased within 15 min and reached maximum values at 30 min (*P* < 0.05 as compared with 0 min group) (Figure 1(e)).

### 3.2. BSZYD Promoted the Expression of VEGF and VEGFR-2 in HEMECs

**Figure 2:**
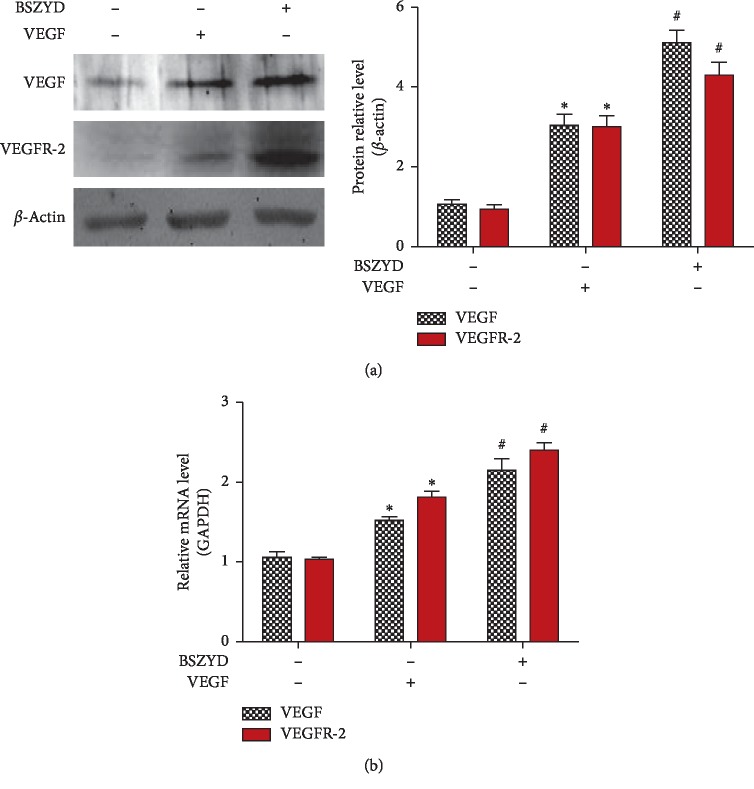
BSZYD promoted the expression of VEGF and VEGFR-2 in HEMECs. (a) HEMECs were incubated in low-serum medium for 24 h and treated with BSZYD for 24 h followed by incubation with or without VEGF (40 ng/mL) for 12 h VEGF and VEGFR-2 levels were determined with western blotting using specific antibodies (left panel); densitometric scanning (right panel). Values are expressed as means ± SD from three independent experiments. ^*∗*^*P* < 0.05 as compared with the control group; ^#^*P* < 0.05 as compared with the VEGF group. (b) VEGF and VEGFR-2 mRNA level analysis with real-time PCR. Values are expressed as mean ± SD from three independent experiments. ^*∗*^*P* < 0.05 as compared with the control group; ^#^*P* < 0.05 as compared with VEGF group.

BSZYD could improve pregnancy rate, but the underlying mechanism is unclear. We observed the effect of BSZYD on HEMECs. After preincubation with BSZYD, HEMECs were treated or untreated with VEGF. Western blot analysis was performed and BSZYD treatment was found to increase the expression of VEGF and VEGFR-2 as compared with the untreated group (*P* < 0.05). Furthermore, the expression levels of VEGF and VEGFR-2 were higher in BSZYD group than in VEGF group (*P* < 0.05) (Figure 2(a)). As shown in Figure 2(b), similar results were observed with RT-qPCR analysis.

### 3.3. BSZYD Induced Angiogenesis and Activated the MAPK Signaling Pathway in HEMECs

**Figure 3:**
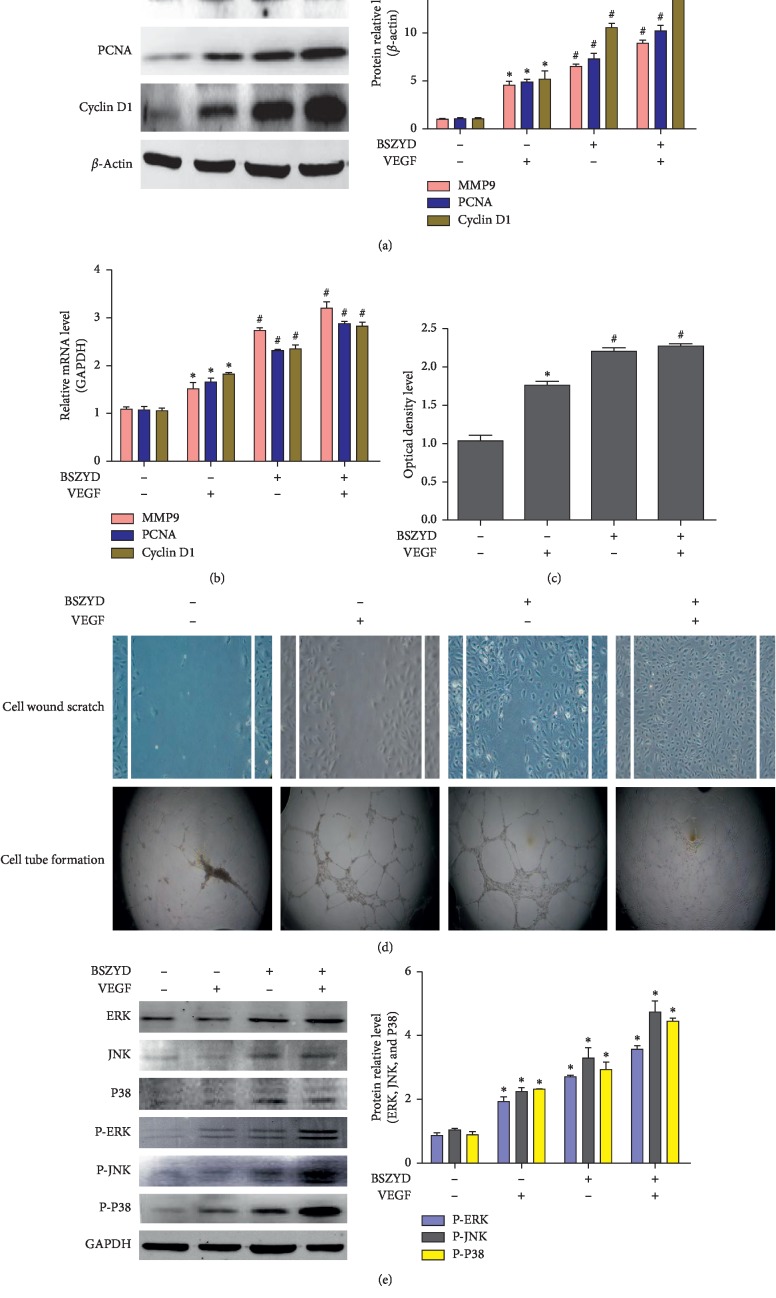
BSZYD induced HEMEC angiogenesis and activated the MAPK signaling pathway. (a) HEMECs were incubated in low-serum medium for 24 h and treated with BSZYD, followed by incubation with or without VEGF (40 ng/mL). MMP9, PCNA, and cyclin D1 levels were determined with western blotting (left panel); densitometric scanning (right panel). Values are expressed as mean ± SD from three independent experiments. ^*∗*^*P* < 0.05 as compared with the control group; ^#^*P* < 0.05 as compared with the VEGF group. (b) The expression levels of *MMP9*, *PCNA*, and *cyclin D1* were determined with real-time PCR. Values are expressed as mean ± SD from three independent experiments. ^*∗*^*P* < 0.05 as compared with the control group; ^#^*P* < 0.05 as compared with the VEGF group. (c) The absorbance from four groups for CCK-8 assay. Values are expressed as mean ± SD from three independent experiments. ^*∗*^*P* < 0.05 as compared with the control group; ^#^*P* < 0.05 as compared with the VEGF group. (d) Images show HEMEC scratch-wound and tube formation assay results. Magnification, ×100. (e) Phospho-ERK, phospho-JNK, and phospho-P38 levels were determined with western blotting using specific antibodies (left panel); densitometric scanning (right panel). Values are expressed as the mean ± SD from three independent experiments. ^*∗*^*P* < 0.05 as compared with the control group; ^#^*P* < 0.05 as compared with the VEGF group.

As proliferation and migration of cells is important for angiogenesis, we evaluated the effects of BSZYD on HEMEC proliferation and migration. After treatment with BSZYD, HEMECs were incubated with or without VEGF. Western blot analysis was performed to detect the expression of PCNA, cyclin D1, and MMP9. In comparison with the control group, VEGF, BSZYD, and BSZYD + VEGF treatment groups showed a significant increase in the expression levels of PCNA, cyclin D1, and MMP9 (*P* < 0.05). The expression levels of PCNA, cyclin D1, and MMP9 in BSZYD and BSZYD +VEGF treatment groups were higher than those reported in VEGF treatment group (*P* < 0.05) (Figure 3(a)). Similar results were observed for RT-qPCR analysis (Figure 3(b)). We also performed CCK-8 assay and found that the absorbance value was significantly higher in the groups treated with BSZYD and BSZYD + VEGF than in the group treated with VEGF (*P* < 0.05) (Figure 3(c)). The scratch-wound assay was performed to determine the effects of BSZYD on HEMEC migration ability. As shown in Figure 3(d), wound healing was significantly faster in BSZYD and BSZYD + VEGF treatment groups than in the untreated cells. Endothelial cell tube formation assay results showed that BSZYD promoted the formation of endothelial cell tube (Figure 3(d)). We examined the expression of MAPK signaling pathway molecules following BSZYD treatment. Western blot analysis showed that the expression levels of p-ERK, p-JNK, and p-P38 proteins in VEGF, BSZYD, and BSZYD + VEGF treatment groups were significantly higher than those in the control group (*P* < 0.05). Thus, BSZYD could activate the MAPK signaling pathway (Figure 3(e)).

### 3.4. BSZYD Induced HEMEC Angiogenesis via MAPK Signaling Pathway

**Figure 4:**
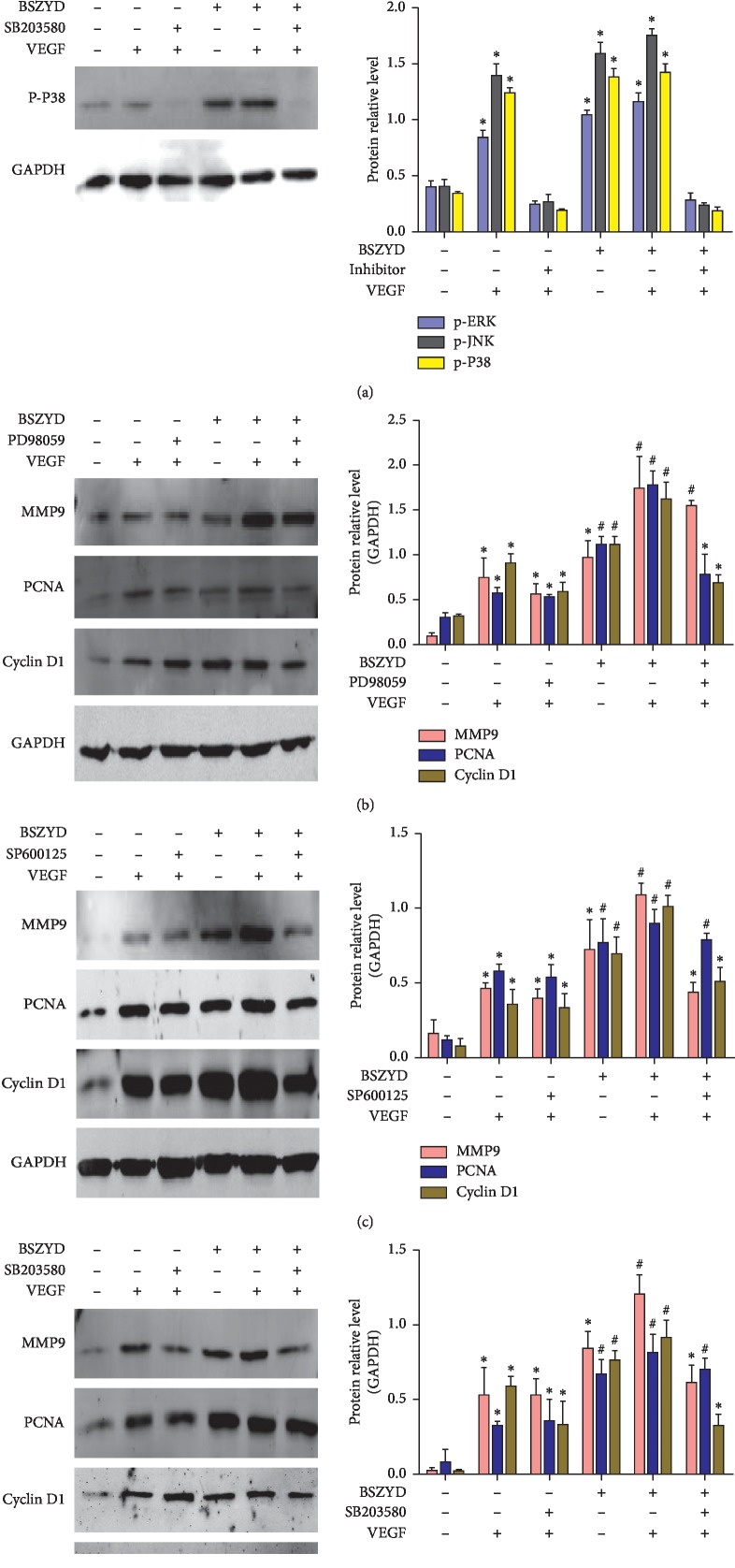
BSZYD induced HEMEC angiogenesis via the MAPK signaling pathway. (a) After incubation in low-serum medium for 24 h, HEMECs were treated with BSZYD, followed by incubation with or without MAPK signaling pathway inhibitors for 2 h. The cells were treated with or without VEGF (40 ng/mL) for indicated times points. Phospho-ERK, phospho-JNK, and phospho-P38 levels were determined with western blotting using specific antibodies (left panel); densitometric scanning (right panel). Values are expressed as mean ± SD from three independent experiments. ^*∗*^*P* < 0.05 as compared with the control group; ^#^*P* < 0.05 as compared with VEGF group. (b-d) MMP9, PCNA, and cyclin D1 levels were determined with western blotting (left panel). Densitometric scanning (right panel). Values are expressed as means ± SD from three independent experiments. ^*∗*^*P* < 0.05 as compared with the control group; ^#^*P* < 0.05 as compared with the VEGF group.

As BSZYD is responsible for angiogenesis and MAPK signaling pathway activation in HEMECs, we examined whether BSZYD induces HEMEC angiogenesis via the MAPK signaling pathway. After incubation with or without BSZYD, HEMECs were treated with the inhibitor of MAPK signaling pathway (PD98059, SP600125, or SB203580), followed by incubation with VEGF. Western blot analysis was performed to detect the expression of the molecules involved in MAPK signaling. In comparison with the control group, VEGF, BSZYD, and BSZYD + VEGF treatment groups showed a significant increase in the protein expression levels of p-ERK, p-JNK, and p-P38 (*P* < 0.05); however, we failed to notice any significant change in the groups treated with inhibitor + VEGF and BSZYD +inhibitor + VEGF (Figure 4(a)). Thus, BSZYD could activate the ERK, JNK, and P38 signaling pathways. In comparison with the BSZYD treatment group, the BSZYD +PD98059 +VEGF treatment group showed a significant reduction (*P* < 0.05) in the protein expression of PCNA and cyclin D1 (Figure 4(b)). The protein expression levels of cyclin D1 and MMP9 in the BSZYD + SP600125 + VEGF and BSZYD + SB203580 + VEGF treatment groups were lower than those in BSZYD group (*P* < 0.05) (Figures 4(c) and 4(d)). These results indicated that BSZYD promotes cyclin D1 expression through the ERK, JNK, and P38 pathways, induces the expression of PCNA through the ERK pathway, and increases MMP9 expression through the JNK and P38 pathways in HEMECs.

## 4. Discussion

Here, we investigated the effects of BSZYD on endometrial receptivity through the evaluation of the proliferation and migration of endothelial cells. As a result, we found that BSZYD could induce the expression of VEGF and VEGFR-2, promote HEMEC proliferation and migration, and mediate angiogenesis. These effects were mediated by the activation of the MAPK signaling pathway.

The controlled ovarian hyperstimulation (COH) protocols, particularly using GnRH analogs, are known to suppress Hoxa11, Meis1, Ctnnb1, and Cdh1 expression in mouse endometrium during the peri-implantation period and impair endometrial receptivity [26]. COH used in IVF is related to lower implantation rates per embryo transferred as compared to natural cycles employed in ovum donation, suggestive of suboptimal endometrial development [27]. Studies have also highlighted the modification of COH regimen to construct an endometrium similar to that under natural cycle in terms of morphology and function, as an attempt to improve pregnancy outcomes [28–30]. As a complementary treatment, TCM may be combined with COH during the IVF cycle to serve as a new strategy to improve the outcome of IVF. In previous studies, we have shown that BSZYD could increase the endometrium thickness in patients with repeated failed implantation of IVF-ET and in superovulation rats, promote the development and formation of pinopodes, and increase the angiogenesis of the endometrium [31, 32]. Also, our study showed that BSZYD can improve the E2 expression in COH mice, which lead to higher pregnancy rate. Meanwhile, BSZYD increases ER expression in HEMECs (data not shown). These results suggest that BSZYDs have estrogen-like activity, which can increase VEGF expression and promote angiogenesis. BSZYD contains nine types of herbs that are effective in improving endometrial receptivity through a multicompound reaction. As shown in Table 1, the phytochemical components of nine Chinese medicines are mainly flavonoids, polysaccharides, and organic acids, and these active principles exhibit wide pharmacological actions on the blood system, immune system, endocrine system, and reproduction system. For example, flavonoids are widely found in plants and berries, they are a strong antioxidant that can effectively scavenge ROS in the body, improve blood circulation, stabilize vascular collagen, and inhibit the exudation of inflammatory enzymes, improving wound healing and pain relief [33, 34]. And flavonoids have estrogen-like activities. Estrogens are steroid hormones exhibiting a broad range of physiological activities, which are important for improving the uterus state and increasing the conception rate [35]. Studies have found that TCM polysaccharides have multiple biological activities, can regulate the immunity by their influence on the secretion of cytokines, possess antioxidative abilities. TCM polysaccharides also possess hepatoprotective, hematopoietic, growth-promoting, antiatherosclerotic activities, and so on [36]. Saponins, their biosynthetic intermediates and derivatives, have been ascribed a number of pharmacological activities, most notably permeabilization of cell membranes, antiplatelet aggregation, immunomodulatory potential, and lowering of serum cholesterol [37]. The chemical components can directly or indirectly promote angiogenesis, which leads to improve the uterus state and increase the conception rate.

Previous studies have demonstrated that angiogenesis is fundamental to the development of a receptive endometrium that permits implantation in primates and humans [38]. VEGF expressed in the endometrium could regulate vascular development, and hence, VEGF inhibitors are used to achieve contraception [38]. Endometrial angiogenesis runs through the complete menstrual cycle. Angiogenesis begins in the proliferative phase and the spiral arteries of the intima undergo prolonged and slow proliferation in the early proliferative phase [39, 40]. These vessels form complex subepithelial capillaries in the late proliferative and early secretory phases. The proliferation of angiogenesis is accelerated from the clump to the middle and late stages of secretion, and the blood flow of the vascular network is maximized. Extensive angiogenesis is an integral part of development of the receptive endometrium [41]. HEMECs are located in the inner mucous membrane of the uterus known as the endometrium. HEMECs are involved in endometrial angiogenesis during the menstrual cycle with the rapid growth and shedding of the endometrium. Cultured HEMECs are a useful model to elucidate the mechanisms of normal angiogenesis and develop treatments for female reproductive disorders. To explore the mechanism of BSZYD promoting angiogenesis, which improve the endometrial receptivity, we choose HEMECs as model system that is more specific than HUVECs.

PCNA is known to regulate cell proliferation, apoptosis, DNA damage repair, and cycle progression [42]. PCNA is overexpressed during peri-implantation and is involved in the reconstruction of the endometrium [43]. Cyclin D1 is also a key regulator of cell proliferation. In endometrial hyperplasia, angiogenesis is closely related to the increase in the expression of cyclin D1; hence, it may be considered as a significant marker of disease prognosis [44]. MMP9 is highly expressed during the embryo implantation window and participates in the degradation and remodeling of the extracellular matrix during the formation and invasion of endometrial spiral arteries [45, 46]. Our study shows that BSZYD induces the expression of PCNA, cyclin D1, and MMP9 in HEMECs (Figures 2 and 3), leading to HEMEC angiogenesis.

**Figure 5:**
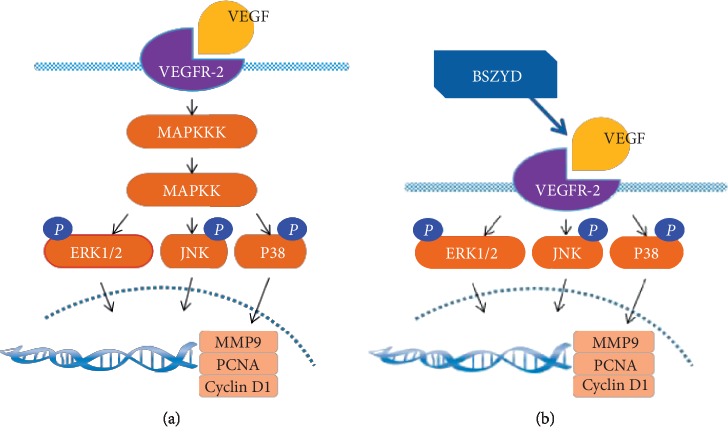
Proposed model for the effects of BSZYD on HEMEC angiogenesis through the VEGFR-2-mediated regulation of the MAPK signaling pathway.

The MAPK family is closely related to the proliferation and migration of vascular endothelial cells and regulates the expression of transcription factors and genes. Blockade of the MAPK signaling pathway may lead to the downregulation in the expression of MMP9, PCNA, and cyclin D1 [47–49]. We found that BSZYD could activate the MAPK (ERK, JNK, P38) signaling pathway (Figure 5).

The MAPK pathways are activated by extracellular and intracellular stimuli, including peptide growth factors, cytokines, hormones, and various cellular stressors. It has been reported that VEGF and VEGFR-2 play a critical role in the regulation of vascular endothelial cell angiogenesis [50, 51]. VEGF and VEGFR-2 form a complex that activates the MAPK signaling pathway (Figure 1). MAPK kinase kinase (MAPKKK)-MAPK kinase (MAPKK)-MAPK (ERK, P38, JNK) transmits upstream signals to downstream response molecules through sequential phosphorylation, finally regulating the expression of specific genes (such as those encoding PCNA, cyclin D1, and MMP9) [52] (Figure 5). BSZYD could promote the expression of VEGF and VEGFR-2 to activate the MAPK signaling pathway (Figure 5). Our study shows that VEGFR-2 was responsible for the expression of PCNA, cyclin D1, and MMP9 upon induction by BSZYD (Figures 2 and 3). Furthermore, we examined the effects of ERK, JNK, and P38 inhibitors on the MAPK signaling pathways induced by BSZYD. As a result, we found that ERK, JNK, and P38 inhibitors blocked the BSZYD-induced ERK, JNK, and P38 signaling pathways and abolished the upregulation in PCNA, cyclin D1, and MMP9 expression mediated by BSZYD. Thus, the ERK, JNK, and P38 signaling pathways play a key role in the effect of BSZYD by promoting HEMEC angiogenesis (Figure 4). BSZYD promoted the expression of cyclin D1 through the ERK, JNK, and P38 pathways, induced PCNA expression through the ERK pathway, and increased MMP9 levels through JNK and P38 pathways. Therefore, BSZYD-induced angiogenesis may be mediated via multiple pathways. The VEGF-VEGFR-2-mediated upregulation in the expression of PCNA, cyclin D1, and MMP9 is one of the mechanisms underlying the angiogenic effects of BSZYD.

## 5. Conclusions

In summary, BSZYD, as a complementary treatment, could provide unique therapeutic advantages by improving endometrial receptivity via multiple pathways. Promotion of VEGF-VEGFR-2 to activate the MAPK signaling pathway is one of the mechanisms underlying the angiogenesis effects of BSZYD. Therefore, further studies are warranted to completely confirm the effects of BSZYD.

## Figures and Tables

**Table 1:** Information list of the 9 herbs of BSZYD.

Chinese medicine	Family	Chinese medicine code	Weight (g)	Phytochemical fractions	Pharamacological activity
*Rehmannia glutinosa*	Scrophulariaceae	A-J-018	20	Iridoids, saccharides, amino acid, inorganic ions [12]	Immune enhancement, regulation of the endocrine system, antisenescence, protective effects on blood system, antitumor effects [12]

*Angelica sinensis*	Apiaceae lindl.	A-J-014	10	Flavonoids, amino acids, trace elements, vitamins and volatile oils, phthalides, organic acids, polysaccharides [13]	Antiblood deficiency, hematopoietic activities, antiinflammatory activity, antifibrotic action, antispasmodic activity, antioxidant activities, and neuroprotective action, cardio- and cerebrovascular effects, antitumor [13]

*Dioscorea oppositifolia*	Dioscoreaceae	A-J-037	15	Yam polysaccharides, diosgenin, adenosine, and arbutin [14–17]	Estrogen-like effects, hypoglycemic effects, antioxidant and antitumor activities, immunomodulatory activity, enhancing cognitive function [14–17]

*Cornus officinalis*	Cornaceae	A-G-075	15	Flavonoids, triterpenes, tannins, saccharides, monoterpenes and sesquiterpenes, iridoids, and essential oils [18]	Hepatic and renal protection, antidiabetes activity, cardioprotection, antioxidation, neuroprotection, antitumor activity, antiinflammation, analgesic effects, antiaging activity, antiamnesia, antiosteoporosis, immunoregulation [18]

*Lyeium chinense*	Solanaceae	A-G-001	12	Polysaccharides, glycerogalactolipids, phenylpropanoids, coumarins, lignans,flavonoids, and alkaloids [19]	Improving immune functions, antioxidative and antiaging, anticancer effect, antifatigue effect, antiviral effect, hepatoprotective effect, hypoglycemic effect, hypolipidemic effect [20]

*Epimedium brevicornu*	Berberidaceae	A-Q-043	10	Epimedins, icariin, flavonoids, and phenolic [21, 22]	Anti-inflammatory activity, regulation of the brain/spinal cord/bone axis, antioxidant activities, improve the ovarian endocrine function [21, 22]

*Astragalus membranaceus*	Papilionaceae	A-J-019	10	Flavonoids, saponins, polysaccharides, and amino acids [23]	Immunomodulatory activity, antioxidant activity, antihyperglycemic activity, anti-inflammatory activity, antiviral activity, dilation of the blood vessels [23]

*Placenta hominis*	Hominidae	A-W-043	10	Gonadal hormone, amino acid, placental globulin, fibrinogen activator, kininase, oxytocin, erythropoietin, phospholipids, and polysaccharides [24]	Anti-infective effect, hormone-like action, enhancing immune function, promoting wound healing [24]

*Cyperus rotundus*	Cyperaceae	A-J-057	10	Essential oils, phenolic acids, ascorbic acids, and flavonoids [25]	Antiandrogenic, antibacterial, anticancerous, anticonvulsant, antidiabetic, antidiarrheal, antigenotoxic, anti-inflammatory, antilipidemic, antimalarial, antimutagenic, antiobesity, antioxidant, antiuropathogenic, hepatoprotective, cardioprotective, neuroprotective, nootropic agent [25]

**Table 2:** Quantitative polymerase chain reaction primer sequences.

Gene	Primer sequence 5′-3′	Amplicon
VEGF	F: 5′-GGAGGAGGGCAGAATCATCA-3′	247 bp
	R: 5′-CTTGGTGAGGTTTGATCCGC-3′	

VEGFR-2	F: 5′-TTACTTGCAGGGGACAGAGG-3′	170 bp
	R: 5′-TTCCCGGTAGAAGCACTTGT-3′	

PCNA	F: 5′-GCTCTTGTTCCCTGGATG-3′	185 bp
	R: 5′-TTTGGCACCCTCACTTTC-3′	

CyclinD1	F: 5′-CCCTCGGTGTCCTACTTCAA-3′	219 bp
	R: 5′-CTTAGAGGCCACGAACATGC-3′	

MMP9	F: 5′-GAGTTCCCGGAGTGAGTTGA-3′	225 bp
	R: 5′-AAAGGTGAGAAGAGAGGGCC-3′	

GAPDH	F: 5′-CACATCGCTGAGACACCATG-3′	198 bp
	R: 5′-TGACGGTGCCATGGAATTTG-3′	

## Data Availability

The data used to support the findings of this study are available from the corresponding author upon request.

## Supplementary Materials

Supplementary Materials Figure 1: BSZYD can increase the expressions of ER in HEMECs. HEMECs were incubated in low-serum medium for 24 h, followed by medium containing BSZYD for 24 h, then treated with E2 (10^–7^ M) or not for 12 h. Total protein lysates were collected from HEMECs and immunoblotted with anti-ER and GAPDH antibodies. BSZYD increases ER expression in HEMECs (Figure 1). This result suggests that BSZYD have estrogen-like activity, which can increase VEGF expression and promote angiogenesis.
