# Supplementary material for: Bu Shen Zhu Yun Decoction Improves Endometrial Receptivity via VEGFR-2-Mediated Angiogenesis
Source: Evid Based Complement Alternat Med. 2019 Dec 31;2019:3949824. doi: 10.1155/2019/3949824 (PMC7011400; doi:10.1155/2019/3949824)
Supplement: Supplementary Materials — Figure 1: BSZYD can increase the expressions of ER in HEMECs. HEMECs were incubated in low-serum medium for 24 h, followed by medium containing BSZYD for 24 h, then treated with E2 (10–7 M) or not for 12 h. Total protein lysates were collected from HEMECs and immunoblotted with anti-ER and GAPDH antibodies. BSZYD increases ER expression in HEMECs (Figure 1). This result suggests that BSZYD have estrogen-like activity, which can increase VEGF expression and promote angiogenesis. [file 3949824.f1.docx]

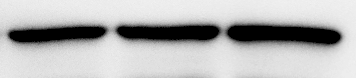

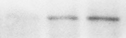


E_2_ - - +

BSZYD - + +

ER

GAPDH

Figure 1. BSZYD can increase the expressions of ER in HEMECs.

HEMECs were incubated in low-serum medium for 24 h, followed by medium containing BSZYD for 24 h, then treated with E2 (10^-7^ M) or not for 12 h. Total protein lysates were collected from HEMECs and immunoblotted with anti-ER and GAPDH antibodies.

BSZYD increases ER expression in HEMECs (Fig. 1). This result suggests that BSZYD have estrogen-like activity, which can increase VEGF expression and promote angiogenesis.
